# Cancer screening among migrants in an Australian cohort; cross-sectional analyses from the 45 and Up Study

**DOI:** 10.1186/1471-2458-9-144

**Published:** 2009-05-15

**Authors:** Marianne F Weber, Emily Banks, David P Smith, Dianne O'Connell, Freddy Sitas

**Affiliations:** 1 Cancer Epidemiology Research Unit, Cancer Council NSW, PO Box 572, Kings Cross 1340, NSW, Australia; 2 National Centre for Epidemiology and Population Health, The Australian National University, Acton ACT 0200, Australia

## Abstract

**Background:**

Limited evidence suggests that people from non-English speaking backgrounds in Australia have lower than average rates of participation in cancer screening programs. The objective of this study was to examine the distribution of bowel, breast and prostate cancer test use by place of birth and years since migration in a large population-based cohort study in Australia.

**Methods:**

In 2006, screening status, country of birth and other demographic and health related factors were ascertained by self-completed questionnaire among 31,401 (16,126 women and 15,275 men) participants aged 50 or over from the 45 and Up Study in New South Wales.

**Results:**

35% of women and 39% of men reported having a bowel cancer test and 57% of men reported having a prostate specific antigen (PSA) test, in the previous 5 years. 72% of women reported having screening mammography in the previous 2 years. Compared to Australian-born women, women from East Asia, Southeast Asia, Continental Western Europe, and North Africa/Middle East had significantly lower rates of bowel testing, with odds ratios (OR; 95%CI) ranging from 0.5 (0.4–0.7) to 0.7 (0.6–0.9); migrants from East Asia (0.5, 0.3–0.7) and North Africa/Middle East (0.5, 0.3–0.9) had significantly lower rates of mammography. Compared to Australian-born men, bowel cancer testing was significantly lower among men from all regions of Asia (OR, 95%CI ranging from 0.4, 0.3–0.6 to 0.6, 0.5–0.9) and Continental Europe (OR, 95%CI ranging from 0.4, 0.3–0.7 to 0.7, 0.6–0.9). Only men from East Asia had significantly lower PSA testing rates than Australian-born men (0.4, 0.3–0.6). As the number of years lived in Australia increased, cancer test use among migrants approached Australian-born rates.

**Conclusion:**

Certain migrant groups within the population may require targeted intervention to improve their uptake of cancer screening, particularly screening for bowel cancer.

## Background

In New South Wales (NSW), Australia's most populous state, 31% of the population aged 45 years or older in 2006 were born outside of Australia [1]. Migration policies in Australia are complex, have changed over time and vary from a selection of skilled migrants using a points system to small groups of refugees accepted on humanitarian grounds [2]. People born in the United Kingdom (UK) are the largest group of migrants nationally (1.1 million persons in 2007), followed by those born in New Zealand (463,300), China (281,000), Italy (225,100) and India (199,700) [3]. A large number of migrants from Europe arrived after the Second World War, whereas many migrants from Asia, North Africa and the Middle East arrived in the 1970's and have increased in number since then [2]. Focusing disease prevention programs in non-mainstream language groups is an international and local challenge and the purpose of this study was to identify migrant groups in Australia that may not be receiving the health care they need. Cancer screening participation is known to vary by demographic factors and limited evidence suggests that people from non-English speaking backgrounds in Australia have lower than average rates of participation in cancer screening programs [4-6]. We used a large cohort study in NSW to investigate variation in the use of bowel cancer tests (faecal occult blood testing – FOBT, colonoscopy, or sigmoidoscopy), mammography, and prostate specific antigen (PSA) tests by migrant group. The objective was to compare screening participation or testing across migrant groups and according to time since migration.

## Methods

### Study Population

The 45 and Up Study is a population-based cohort study of people aged 45 and over in NSW[7] Participants were randomly sampled from Medicare Australia, Australia's universal health insurance system, which includes all citizens and permanent residents of Australia, some temporary residents and refugees. Residents in regional and remote areas and those aged 80 and over were over-sampled by a factor of two. Participants completed a mailed self-administered questionnaire and consent form (available at ) [8]. The participation rate was 18%. We report the analysis of data from 31,401 people aged 50 and over who completed the questionnaire between February 2006 and June 2006. We chose the lower age limit of 50 years because screening for bowel and breast cancer is not recommended for people younger than 50 if they are at normal risk.

The 45 and Up Study has been approved by the University of New South Wales Human Research Ethics Committee and the Cancer Council New South Wales Ethics Committee.

### Ascertainment of screening use

A self reported history of bowel and breast cancer screening was ascertained from the questions, "Have you ever been screened for colorectal (bowel) cancer/been for a breast screening mammogram?". History of PSA testing was ascertained from the question, "Have you ever had a blood test ordered by your doctor to check for prostate disease? (PSA test)". Respondents were also asked to indicate how long ago (in years) they had used each test type.

Participants reporting a personal history of bowel cancer were excluded from bowel screening analyses as these individuals would be undergoing more frequent surveillance. Likewise, analyses of breast screening excluded women who reported a history of breast cancer and analyses of PSA testing excluded men with a personal history of prostate cancer.

**Figure 1:**
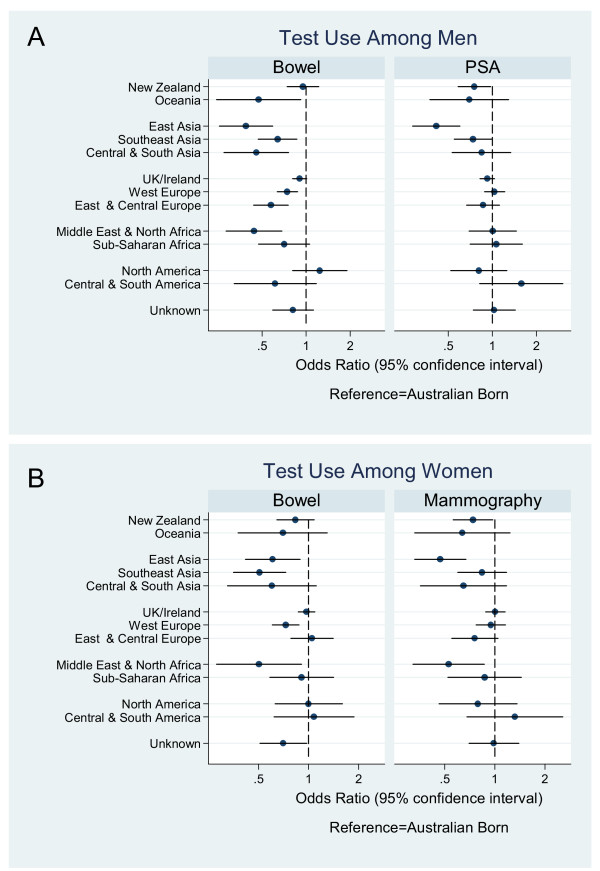
**Test use by place of birth. **A) Bowel and prostate specific antigen (PSA) test use in men by place of birth relative to Australian-born. B) Bowel test and mammogram use in women by place of birth relative to Australian-born. Odds ratios are adjusted for age, education, family history of cancer and health insurance status.

Place of birth was grouped according to a modified version of that used in the Global Burden of Disease Study (see Figure 1) [9].

### Analyses

We compared the proportions of participants who undertook bowel cancer or PSA tests within the last 5 years, and mammograms within the last 2 years by place of birth, time since migration and whether or not English was spoken at home.

The prevalence of test use within the cohort was weighted for age (according to the NSW population in 2006) [1] and region of residence using the distribution of the NSW population according to the Accessibility/Remoteness Index of Australia (ARIA+; 2001) [10].

Odds ratios and 95% confidence intervals for being tested for each type of cancer by migrant factors were estimated using logistic regression. Each model was stratified by gender and adjusted for age (4 levels: 50–59, 60–69, 70–79, 80+), highest level of education achieved (none, 10 years of schooling – 'School Certificate', 12 years of schooling – 'Higher School Certificate'/Trade/Diploma, university degree), family history of relevant cancer type (bowel/breast/prostate, other, none), private health insurance status (yes/no) and place of residence (ARIA+ 2001). The final models did not include place of residence because it made no difference to the odds ratios. Years lived in Australia for migrants was analysed as a continuous variable with an additional adjustment for place of birth. All models included a 'missing/unknown' level for each covariate.

## Results

Of those in the cohort aged 50 years or over, 26.0% of men and 22.5% of women were migrants (24.2% of the cohort). The distribution of demographic factors by place of birth for men and women are shown in Tables one and two, respectively in Additional file 1

Of the 15,275 men aged 50 years or over, 1,090 reported having been diagnosed with prostate cancer and were excluded from analyses on PSA testing. 322 men reported ever having bowel cancer and were excluded from analyses on bowel cancer testing. Of the 16,126 women aged 50 years or over, 226 reported a previous diagnosis of bowel cancer and were excluded from analyses on bowel cancer testing and 966 women reported a previous diagnosis of breast cancer and were excluded from analyses on mammography.

Table three in Additional file 1 shows the distribution of test use by Australian-born versus migrant participants. Overall, 39% of men reported having a test for bowel cancer in the last 5 years and 57% reported having a PSA test. The prevalence of bowel cancer test use among men, weighted for age and region of residence, was 39%, and for PSA test use was 56%. Thirty five percent of women reported having a test for bowel cancer within the last 5 years and 72% reported having a mammogram in the last 2 years. The age- and region- weighted prevalence of bowel test use for women was 34% and for mammography was 68%.

**Figure 2:**
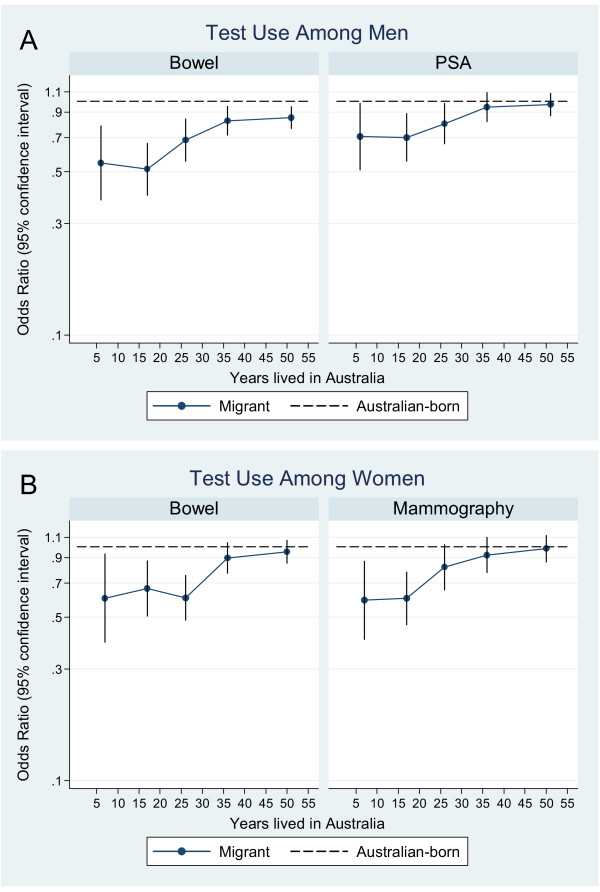
**Test use by years lived in Australia.** A) Odds ratios for bowel and prostate specific antigen (PSA) test use in migrant men by years lived in Australia, compared to use in Australian-born men. B) Odds ratios for bowel test and mammogram use in migrant women by years lived in Australia, compared to use in Australian-born women. Odds ratios are plotted at the median value for each category of time since migration: 1–10 years, 11–20 years, 21–30 years, 31–40 years, and 40+ years, and are adjusted for age, education, family history of cancer and health insurance status.

After adjusting for age, education, family history of cancer and health insurance status, there was significant variation in the use of bowel cancer tests across migrant groups. Men from Oceania (OR = 0.47, 95% confidence interval 0.24–0.92), East Asia (0.39, 0.25–0.59), Southeast Asia (0.64, 0.47–0.87), South and Central Asia (0.46, 0.27–0.76), Continental Western Europe (0.74, 0.63–0.87), Central and East Europe (0.57, 0.44–0.76) and the Middle East/North Africa (0.44, 0.28–0.68) reported lower levels of bowel test use than Australian-born participants (see Figure 1A). Similarly, men who spoke a language other than English at home were less likely to report using a bowel cancer test than English-only speakers (0.60, 0.52–0.68). However, reported use of bowel cancer tests among migrants approached Australian-born rates as the number of years lived in Australia increased (see Figure 2A). After adjusting for place of birth, the odds of migrant men reporting a bowel cancer test increased by 9% (1.09, 1.03–1.15, *p *= 0.003) with every 10 years lived in Australia, yet remained lower than the Australian-born rate irrespective of years lived in Australia.

There was variation in PSA test use by place of birth (Figure 1A), however only men from East Asia had significantly lower rates of PSA test use than Australian-born men (0.41, 0.29–0.60). Test use increased to Australian-born rates as years lived in Australia increased (Figure 2A). Adjusting for place of birth, the odds of migrant men reporting a PSA test increased by 8% (1.08, 1.03–1.14, *p *= 0.004) with every 10 years lived in Australia. Language spoken at home was associated with reduced use of PSA tests (0.85, 0.75–0.96).

Women born in East Asia (0.61, 0.42–0.89), Southeast Asia (0.51, 0.35–0.73), Continental Western Europe (0.73, 0.60–0.88) and the Middle East/North Africa (0.50, 0.28–0.91) were significantly less likely to report having a test for bowel cancer within the previous 5 years than Australian-born women (see Figure 1B). Across all migrant women, rates of bowel cancer testing approached that of Australian-born women as the number of years since migration increased (see Figure 2B). With every 10 years lived in Australia, the odds of migrant women reporting a test for bowel cancer increased by 11% (1.11, 1.05–1.18, *p *= 0.001), reaching the Australian born rate after around 35 years of living in Australia. Women who spoke a language other than English at home were less likely to report a bowel screening test than those who spoke only English (0.77, 0.68–0.89).

There was significant variation in mammography use by place of birth (Figure 1B) however only women from East Asia (0.47, 0.33–0.67) and the Middle East/North Africa (0.53 0.32–0.86) had significantly lower rates of mammography than Australian-born women. As the number of years lived in Australia increased for migrant women, the odds of reporting a mammogram reached those of Australian-born participants (Figure 2B). However, after adjusting for place of birth, mammogram use was not significantly associated with years lived in Australia (1.06, 0.99–1.13, *p *= 0.08). Language spoken at home was associated with reduced use of mammograms (0.83, 0.72–0.96).

## Discussion

We found that within a large cohort of people aged 50 years and over in Australia, there is variation in the use of tests for bowel, breast and prostate cancer both within and between migrant groups. The odds of bowel cancer test use (FOBT, colonoscopy, or sigmoidoscopy) among participants from non-English speaking backgrounds (primarily Asia and Europe) was significantly lower than Australian-born participants. Indeed, for participants who reported speaking a language other than English at home, the odds of reporting a test for bowel cancer within the previous 5 years was up to 40% lower than for English-only speakers. In contrast, mammography use among women and PSA test use among men, did not vary widely by migrant group. The odds of reporting a mammogram for women born in East Asia and North Africa/Middle East were half those for Australian-born women, and the odds of a man born in East Asia reporting a PSA test were reduced by 60%. Use of bowel and PSA tests by migrants increased with increasing number of years lived in Australia, taking about three decades to approach Australian born rates.

In Australia, a freely available program for bowel cancer screening had not begun at the time of this survey so it is not surprising that bowel cancer test use overall is lower than mammography and PSA test use. Free population screening for breast cancer has been offered to women aged 50–69 since 1992 and has been widely advocated. PSA testing, while not currently recommended as a population-based screening tool for prostate cancer, is subsidised by the government and has had considerable media attention [11,12].

Among women, mammography use did not vary by migrant group as much as bowel test use among women. Nevertheless, women from East Asia and the Middle East/North Africa were significantly less likely than Australian-born women to have had a mammogram within the previous two years and these groups of women make up a substantial proportion of the Australian population (i.e. 11% of the NSW population were born in Asia and the Middle East/North Africa) [1]. Lower than average participation in breast screening programs among ethnic minority groups has also been reported in the USA [13-16], and the breast cancer mortality rate for Asian and Pacific Islander women in the USA increased by 302% between 1980 and 2001 [16]. Trends in breast cancer mortality among women from Asia and North Africa/Middle East in Australia have not been reported, but this would be an important issue to follow-up.

Among men, reported PSA test use did not vary widely by migrant group. Only men from East Asia reported significantly lower PSA test use than Australian-born men. Ethnic variation in PSA test use among migrant groups to the USA have been reported [17,18]. Psychological factors such as fear and knowledge of prostate cancer were associated with test use. To our knowledge, cultural differences in PSA test use have not been widely explored, nevertheless, until there is clear evidence from randomised controlled trials as to whether screening with PSA testing reduces prostate cancer mortality, the implications of a lower than average use of PSA testing are not known.

Bowel cancer testing was significantly lower among migrants from non-English speaking backgrounds than for Australian-born participants. Among cultures in Asia, Oceania, North Africa and the Middle East there may be a lack of awareness about bowel cancer in general. Bowel cancer incidence and mortality rates in Asia are much lower than in Australia [19], and so compared to Australian-born people, migrants from these cultures may have had less exposure to bowel cancer within their communities. Conversely, in most parts of Europe bowel cancer is one of the leading cancer types and incidence and mortality rates are similar to those observed in Australia [19]. It is unclear why migrants from continental Europe in this cohort are less likely to have a test for bowel cancer than Australian-born people, especially since they have similar rates of mammography and PSA testing. Further research to identify cultural and other barriers to bowel screening for migrant Australians is important.

Overall, our results suggest that barriers to bowel cancer screening arising from not having a freely available and accessible program appear to have affected migrants from non-English speaking backgrounds to a greater extent than Australian-born people. However, low rates of participation among those from non-English speaking backgrounds were also reported in the freely available National Bowel Cancer Screening Pilot, suggesting that factors other than cost and accessibility are barriers to participation among these groups [20]. Evidence from both Australia [21-23] and elsewhere [24] suggests that a strong predictor of cancer screening participation is encouragement from health professionals. We observed that participants who reported having a mammogram or PSA test were more likely to also report a bowel test compared with those who did not (Table three in Additional file 1). Indeed, in a previous report, we found that 45 and Up Study participants were more than twice as likely to have a FOBT for bowel cancer if they had also had a mammogram or a PSA test[25] This suggests that bowel screening should be promoted further through existing health networks.

Across all the test types examined here, one group that displayed consistently low rates of cancer screening was participants from East Asia. It is possible that this group of people under-utilise health services in general. Cultural rather than demographic factors are the likely cause of this, as participants from East Asia were in the target age group for cancer screening (50–59 years), were generally well educated (e.g., 44% of East Asian men had a university degree), primarily lived in major cities, and a high proportion had private health insurance. These factors are usually associated with high participation in cancer screening programs [24]. One major barrier to screening for people of Asian descent maybe a lack of understanding regarding the purposes of screening [15,26-29]. In particular, research suggests that some Chinese communities tend to feel that screening for disease is unnecessary if they 'feel well' and do not have symptoms [28,29]. The East Asian community in Australia is large and has been increasing consistently over time [1], so education and awareness regarding cancer screening should be targeted at this group.

Our finding that cancer screening among migrants increases with the number of years since migration has also been reported in studies elsewhere [16,30,31], and is likely to be due to factors related to acculturation. For example, geographic history, language use and fluency, identity, friendship circles and attitudes have been found to reflect the degree of acculturation and are positive predictors of mammography uptake among Asian-American women [31]. In this study, the increase in cancer screening participation with increased years lived in Australia was particularly apparent for bowel screening. One reason for this is that many migrants come from places where the incidence rate of bowel cancer is lower than in Australia and indeed, migrants in NSW have lower rates of bowel cancer than Australian-born residents [32]. Part of the process of acculturation would include an increasing awareness of bowel cancer not only via media and health professionals, but also via community networks.

Cancer screening history in this study was derived from self-report. A meta-analysis of validation studies on self-reported cancer screening use in the USA found that self-reported versus documented history of screening had reasonably high sensitivity (ranging from 0.71 for PSA, to 0.95 for mammography) and specificity (ranging from 0.61 for mammography, to 0.90 for colorectal endoscopy) [33]. However, a specific issue with self-report in this study is that although the questionnaire specifically asked about "screening" there was no information regarding the difference between screening and diagnostic testing. This may have introduced differential measurement error if migrants had a lesser understanding of the concept of 'screening' than Australian-born participants. If this was the case, then a higher proportion of migrants would have mistaken a diagnostic test for a screening test and the differences observed in test uptake for colorectal and breast cancer between migrants and Australian-born participants would have been under-estimated.

Cohort study participants tend to be healthier and more health conscious than non-participants [34]; hence, we expect screening rates to be somewhat higher in the 45 and Up cohort than the general population. For example, the age- and region -adjusted prevalence of bowel cancer test use by women reported here (34%) is higher than that reported in a previous survey of health risk factors in the general population of women aged 50 and over in NSW (24%) [35]. Cohort studies are designed to provide reliable information on the effects of different risk factors on outcomes, rather than population prevalence estimates and are generalisable even when based on highly selected groups [36,37]. Potential bias resulting from the "healthy cohort" effect, if it is present, and the fact that the study questionnaire was available only in English, may have led to conservative results. The proportion of migrants in the cohort with English as a second language was smaller than the proportion in the NSW population [1] suggesting reduced participation among non-English speaking migrants. Migrants with limited skills in English may be more likely than other participants to misunderstand the questionnaire. These issues mean that the relative risks associated with screening behaviour may be underestimated. Caution must therefore be exercised when interpreting negative results. Nevertheless, our findings are similar to those from studies using population-based registry data from the USA and Sweden where reduced rates of mammography and bowel cancer screening were observed among migrants, and in particular, migrants originating from certain places in Asia [14,15,38-40].

NSW has one of the most heterogeneous populations in the world, with almost a third of those aged 45 and over having migrated to Australia. The 45 and Up Study is the largest cohort study in NSW and the results presented here describe significant variation in the use of three cancer screening paradigms across a diverse range of migrant groups using uniform outcome measures. The 45 and Up study also allowed adjustment for important demographic factors related to screening use, as well as family history of cancer and access to health services. Further, this is the first Australian study, and the biggest internationally, to describe PSA test use across different cultural groups.

## Conclusion

It is of general concern that many migrants from non-English speaking backgrounds are less likely to have bowel or breast cancer screening tests than participants who were born locally. Differences in cancer screening use between migrants and locally-born participants were particularly marked for bowel screening test use. However, there is potential for FOBT rates to match those of mammography and PSA testing and so it will be interesting to monitor trends in screening participation within the cohort as the National Bowel Cancer Screening Program becomes more widely available.

## Competing interests

The authors declare that they have no competing interests.

## Authors' contributions

Study conception and design: Weber, Banks, Sitas. Drafting the manuscript: Weber, Banks. Statistical analysis and interpretation: Weber, O'Connell, Banks. Revision of the manuscript for important intellectual content: Sitas, O'Connell, Smith. All authors read and approved the final manuscript.

## Pre-publication history

The pre-publication history for this paper can be accessed here:



## Supplementary Material

Additional file 1 **Weber Tables**. Contains Tables 1 – 3. Click here for file
